# Supplementary material for: Current characteristics of animal rabies cases in Thailand and relevant risk factors identified by a spatial modeling approach
Source: PLoS Negl Trop Dis. 2021 Dec 1;15(12):e0009980. doi: 10.1371/journal.pntd.0009980 (PMC8668119; doi:10.1371/journal.pntd.0009980)
Supplement: S1 Tables — Table A in S1 Tables Cluster analysis of animal rabies cases in Thailand in 2017. Table B in S1 Tables Cluster analysis of animal rabies cases in Thailand in 2018. (PDF) [file pntd.0009980.s001.pdf]

Supplementary material 1

Table A Cluster analysis of animal rabies cases in Thailand in 2017

Cluster	Latitude	Longitude	LLR	P-value	Observed	Expected	ODE	RR
1	13.40219	101.41844	109.6221	0.0000	65	8.31	7.82	8.39
2	15.90570	103.78679	85.5239	0.0000	58	8.41	6.90	7.33
3	6.79327	100.61135	52.0751	0.0000	44	7.81	5.63	5.88
4	13.55822	100.73626	45.4654	0.0000	37	6.33	5.85	6.07
5	14.84798	103.29852	38.0937	0.0000	38	7.81	4.86	5.04
6	16.37512	104.70444	35.9457	0.0000	37	7.81	4.74	4.91
7	15.91246	104.17948	29.0853	0.0000	30	6.33	4.74	4.88
8	16.05330	102.07115	24.8809	0.0000	14	1.68	8.33	8.45
9	12.48540	99.96002	21.3027	0.0000	13	1.68	7.73	7.84
10	13.82008	100.52850	20.3557	0.0000	19	3.66	5.19	5.29
11	7.90665	99.99760	18.5488	0.0001	11	1.38	7.94	8.04
12	7.04737	99.81580	18.3433	0.0001	28	8.01	3.50	3.58
13	14.60672	102.83399	18.2635	0.0001	17	3.26	5.21	5.29
14	14.00121	100.68632	14.5648	0.0034	23	6.73	3.42	3.49
15	14.86741	104.40471	14.2061	0.0046	11	1.78	6.18	6.25
16	16.32462	103.99112	13.8915	0.0061	18	4.55	3.96	4.02
17	13.53557	100.37867	13.4410	0.0120	15	3.36	4.46	4.52
18	14.05362	101.34653	13.2817	0.0130	25	8.21	3.05	3.11
19	16.25537	103.48096	11.6842	0.0280	24	8.31	2.89	2.94

Table B Cluster analysis of animal rabies cases in Thailand in 2018

Cluster	Latitude	Longitude	LLR	P-value	Observed	Expected	ODE	RR
1	15.95921	104.22642	61.3811	0.0000	59	13.37	4.41	4.56
2	7.10404	100.53270	51.4503	0.0000	57	14.29	3.99	4.11
3	15.10861	103.47220	41.0283	0.0000	53	14.75	3.59	3.69
4	13.40219	101.41844	37.2204	0.0000	51	14.75	3.46	3.55
5	12.57652	99.94336	33.8834	0.0000	40	10.45	3.83	3.91
6	15.94461	104.75046	28.7737	0.0000	45	14.13	3.18	3.25
7	6.61205	100.11684	28.4462	0.0000	46	14.75	3.12	3.19
8	15.66308	102.48804	27.9746	0.0000	33	8.60	3.84	3.90
9	14.85940	103.49397	25.2677	0.0000	39	12.14	3.21	3.27
10	14.65118	103.86477	25.0241	0.0000	43	14.29	3.01	3.07
11	16.32006	103.92406	24.4366	0.0000	40	12.90	3.10	3.16
12	15.10907	102.17357	21.4520	0.0000	30	8.76	3.43	3.48
13	12.90793	101.37274	20.7362	0.0000	25	6.61	3.78	3.83
14	13.63541	100.79849	18.3874	0.0001	20	4.92	4.07	4.11
15	16.61073	104.46076	16.3509	0.0005	37	14.29	2.59	2.63
16	14.47810	103.32296	15.3522	0.0012	37	14.75	2.51	2.55
17	13.78196	101.27646	14.9840	0.0017	17	4.30	3.95	3.99
18	15.99923	103.66120	14.6202	0.0024	23	7.22	3.19	3.22
19	9.03408	99.56527	13.5478	0.0066	17	4.61	3.69	3.72
20	14.27139	102.29160	11.5312	0.0350	11	2.46	4.48	4.50
