# Supplementary material for: Current characteristics of animal rabies cases in Thailand and relevant risk factors identified by a spatial modeling approach
Source: PLoS Negl Trop Dis. 2021 Dec 1;15(12):e0009980. doi: 10.1371/journal.pntd.0009980 (PMC8668119; doi:10.1371/journal.pntd.0009980)
Supplement: Attachment — Submitted filename: Response to Reviewers R1.docx [file pntd.0009980.s002.docx]

Dear Editor and reviewers,

We are very grateful for your valuable time used in reviewing our manuscript. We now respond to your comments and suggestions as the following. We hope that the current version would be suitable for publication.

Yours sincerely,

Anuwat Wiratsudakul (corresponding author)

**Reviewer #1:**

In the study “Current characteristics of animal rabies cases in Thailand and relevant risk factors identified by a spatial modeling approach” Wiratsudakul et al. describe the application of GAM model for generating risk maps of rabies using secondary data from Thailand. The article is well written and built on a sound premise and hypothesis. The results are interesting and can be relevant, especially for public health and those interested in applying statistical/computational methods to improve health surveillance. However, there are some major points that need to be clarified before the same could be published.

Comments: at line 121 authors provide the general formula for a GAM model. However, it’s not possible to check which was the final model used to produce the results. Based on figure 2, it seems that the smooth function was used for Main road, Distance to border, Density of Owned Dog, Human Population Density, Density of cattle population and Month of Rabies occurrence. Is this correct? Which function did the authors use for each of these?

Response: Smooth function in GAM was used for continuous variables including Main road, Distance to border, Density of Owned Dog, Human Population Density, Density of cattle population and Month of Rabies occurrence while logit function was used for binary variables including the rabies occurred in owned sub-district in the past month and the rabies occurred in the neighboring sub-district (s) in the past month.

Comments: Authors use the term Spatiotemporal. However, while it is possible to see that the authors are using a kind of connectivity matrix, which I believe was used to lead with spatial auto-correlation, the same is not true for time. Could the authors explain better how to address the problem? Again, I believe that specifying the final model could be very helpful here.

Response: The temporal correlation was considered through the temporal autoregressive term (we included rabies count in the previous month as predictor), which should control for the temporal autocorrelation.

Comments: I believe that table two will be more informative if the authors present point estimates and confidence intervals instead of p-value.

Response: We agree with the reviewer and add lower and upper confidence intervals in the table 2.

Comments: Generally, when you try to set a predictive model, it is common to check which features have the biggest impact on predictions. Could you please provide this information?

Response: We assess the impact of the predictors by adding a table (Table 3) to describe the models using three indicators including AIC, R2, and Deviance explained. As a result, it was found that the rabies that occurred in neighboring sub-district(s) in the past month caused the biggest impact. We address the new finding as “Besides, we found that ‘rabies occurring in neighboring sub-district(s) in the past month’ caused the largest impact (Table 3).”

Comments: Change model set to train set.

Response: We change the model set and the train set and address this in the method; “At the end, we also swapped between the model set and the train set to see the other way around”. Then, we put in the result section; “After swapping the model and the train sets, we found that, in the whole period, the AUC values of the model set indicated a higher accuracy with a mean of 0.807 (range: 0.801-0.812) compared to that of the test sets (mean AUC value = 0.727; range: 0.718-0.735) (Fig. 3B). In the validation of the monthly models, the mean of AUC values in the model sets ranged between 0.690 and 0.848 (Fig. 4C) while that of the test sets ranged between 0.718 and 0.817 (Fig. 4D). The results were not much different from the original tests”.

**Reviewer #2:**

The objectives are clearly articulated with the hypothesis presented. The design of the study is partially appropriate. The analysis of spatio-temporal patterns was compromised due to the short period of time worked by the authors. The population is well described and appropriate for the hypotheses being tested, despite the limitations inherent in the use of secondary data from surveillance systems. Due to the time interval used (2 years), the sample size is insufficient to guarantee adequate power to address the tested hypotheses. In addition, the authors did not present a sample design plan. The statistical analysis used is adequate to support the conclusions. Not describe the necessary ethical authorizations for obtaining animal samples.

The analysis presented are in accordance with the analysis plan, despite the limitations indicated in the study methodology (Short time interval and use of secondary bases from local surveillance systems) The results are presented in a clear and complete manner, despite the lack of information in the initial descriptive analysis, for some variables obtained from the surveillance bases. The figures shown are of sufficient quality.

Comments: The methodological limitations of the manuscript directly impact the quality of the results presented. The authors did not clearly present the limitations of the analysis, such as the incompleteness of the data obtained in the surveillance services, and the short time used in the analyzes, which substantially prevents adequate interpretations of space-time patterns and seasonality.

Response: We acknowledged the incompleteness of our data in the discussion part (in the paragraph before conclusion); “The main limitation of this study was the incompleteness and unavailability of some data. As appeared in the descriptive analysis of rabies cases derived from Thairabies.net, several important variables were unidentified such as ownership (12%), vaccination history (50.4%), and animal-keeping practices (69.1%). In our spatial modeling, some important data, such as vaccine coverage, were unavailable and/or incomplete. This is one of the plausible explanations why low accuracy was found in some of our predictive maps.” We also suggested that “This is one of the plausible explanations why low accuracy was found in some of our predictive maps. The improvement of robust routine data collection is required for better analysis and prediction in future studies”. However, we agree with the reviewer that we missed that point of long-term prediction. We hence added the sentences; “Moreover, we focused on the changes that occurred during the epidemic years and we tried to produce a tool for a short time prediction based on spatial characteristics of a certain area. Therefore, our model did not reflect the long-term space-time patterns and seasonality. A future study working on more longitudinal data is recommended.”

Comments: In addition, the authors do not adequately discuss how the data can be useful to advance the understanding of the topic under study, limiting themselves to bringing local and regional aspects; And public health relevance has been poorly addressed, and could be further developed.

Response: We discuss more as the following; “What we observed from our study would be useful to guide relevant public health policies. For example, in public education, we should ask people to pay more attention to the owned dogs rather than only strays. Besides, annual canine rabies vaccination should be rigorously emphasized among dog owners. In terms of disease control, veterinary authorities should focus more on repeated outbreak areas with densely human populations as the risk of recurrence is high. Ultimately, our modelling framework is readily integrable to a real-time surveillance system to produce a timely predictive model resulting in a better preparedness intervention.”

Comments: Include information on ethical procedures related to obtaining animal samples (if available). To address in the discussion global aspects related to the findings found in the study and bring the relevance of the conclusions to the field of public health.

Response: We add sentences regarding ethical procedures; “We only used secondary data supplied through the system. No human or animal subjects were involved. The study thus required no human or animal ethical approvals. However, the study designs and protocols used were approved by the Department of Livestock Development, Thailand”. We discuss our findings in more general terms to make it more readable; “For example, in public education, we should ask people to pay more attention to the owned dogs rather than only strays. Besides, annual canine rabies vaccination should be rigorously emphasized among dog owners. In terms of disease control, veterinary authorities should focus more on repeated outbreak areas with densely human populations as the risk of recurrence is high. Ultimately, our modelling framework is readily integrable to a real-time surveillance system to produce a timely predictive model resulting in a better preparedness intervention”.

Comments: Original study with relevance for the area, and of interest to researchers, professionals and policy makers specific to animal and human rabies. Apparently, there is no potential interest for researchers or professionals outside the area. Partially rigorous methodology, with conclusions justified by the evidence presented, but with important limitations in the methodology that directly impact the interpretation of the results presented. There was no mention in the manuscript of ethical approvals in the animal field.

Response: We acknowledge that our target readers are policymakers on rabies issues. Others working in different fields may also get some indirect benefits such as the techniques used in this study which is applicable in other diseases. We add some sentences regarding ethical approval; “We only used secondary data supplied through the system. No human or animal subjects were involved. The study thus required no human or animal ethical approvals. However, the study designs and protocols used were approved by the Department of Livestock Development, Thailand”.

Comments: It would be very important to extend the study period for the conclusions about temporal space patterns to become robust. Working with only two years, limits interpretation of the findings, since only "epidemic" years were included. This is also reflected in the active / passive surveillance service, which, theoretically, may be more attentive and agile in recent years, due to the significant increase in diagnosed / reported cases.

Response: We definitely agree with the reviewer that extending the study period would provide a better spatio-temporal pattern and seasonality. However, the reason that we worked only on the recent ‘epidemic’ years was that we can see more clearly the immediate trend of the epidemic as well as relevant spatial factors. In this study, we intended to produce a prediction tool using the immediate outbreak, in the previous month, to estimate the likelihood of having the immediate future outbreak based on different spatial characteristics of an area. Indeed, the Department of Livestock Development of Thailand, in which we are in the working group, is now developing a real-time prediction tool based on the continuous flow of data in our database and we will apply this method in our system soon. We therefore would like to be sure that our method is valid by peer-reviewing in a renowned journal like PLOS Neglected Tropical Diseases. The comments and suggestions of the reviewers are very helpful for our work. To address this point, we add in the paper: “Ultimately, our modelling framework is readily integrable to a real-time surveillance system to produce a timely predictive model resulting in a better preparedness intervention”.

Regarding the increased surveillance services during epidemics, we added sentences in the paragraph of limitations under the discussion section; “Nevertheless, the data we used here were based on the epidemic years in which the surveillance activities may increase due to higher number of diagnosis and reported cases. The data on non-epidemic years should be also considered in the future study”.

Comments: In the introduction, the authors provide a historical overview of notifications of animal and human rabies in the studied territory. It is suggested to include in the analysis a broader period of time, for example, 10 years, so that the historical series includes a significant period of time, which better reflects the reality of rabies in the territory.

Response: As mentioned in the response earlier, our objective is to develop a rabies prediction tool based on spatial features of a certain area and use it in our national surveillance activities. However, the longitudinal study suggested by the reviewer is also useful for our country. We are very grateful and we would consider this approach in our future study. To address this point, we add in the discussion; “Moreover, we focused on the changes that occurred during the epidemic years and we tried to produce a tool for a short time prediction based on spatial characteristics of a certain area. Therefore, our model did not reflect the long-term space-time patterns and seasonality. A future study working on more longitudinal data is recommended”.
